# Risks of malignant lymphoma in rheumatoid arthritis patients receiving methotrexate-alone and in combination therapy compared with the general population: A study based on a Japanese medical claims database 

**DOI:** 10.5414/CP204372

**Published:** 2023-08-14

**Authors:** Ryo Inose, Arisa Nakamura, Rina Omi, Shujiro Takeno, Yuichi Muraki

**Affiliations:** 1 Department of Clinical Pharmacoepidemiology, Kyoto Pharmaceutical University, Kyoto, and; 2 Real World Data Consulting, IQVIA Solutions Japan, Tokyo, Japan

**Keywords:** methotrexate, biological disease-modifying antirheumatic drugs, malignant lymphoma, working-age population, claims database

## Abstract

Objective: The risk of malignancy in patients with rheumatoid arthritis (RA) treated with methotrexate (MTX) and biological disease-modifying antirheumatic drug (bDMARD) combination therapy is unknown. This study aimed to clarify the incidence of malignancy and the recommended monitoring period in patients receiving this combination therapy. Materials and methods: A retrospective, observational study based on a large Japanese medical claims database was conducted between April 2013 and February 2020. Patients with RA were classified into MTX-alone and combination therapy groups, and the standardized incidence rates (SIR) of malignancy were calculated. The time of onset of malignancy in both groups was calculated. Results: In total, 2,052 patients received MTX-alone and 782 received combination therapy. The incidence of malignant lymphoma was significantly higher with MTX-alone therapy (SIR: 6.09, 95% confidence interval (CI): 1.58 – 10.61) and combination therapy (SIR: 20.86, 95% CI: 8.53 – 33.19) than in the general Japanese population. Furthermore, the combination therapy had a significantly higher risk of malignant lymphoma than the MTX-alone therapy (adjusted odds ratio: 4.27, 95% CI: 1.64 – 11.12). The median time from MTX prescription to the onset of malignant lymphoma was 3.58 years (interquartile range (IQR): 2.00 – 5.34 years) for MTX-alone and 3.42 years (IQR: 1.25 – 4.92 years) for combination therapy. Conclusion: The incidence of malignant lymphoma in the combination therapy group was extensively higher than that in the general Japanese population. Special attention is required for early symptoms of malignant lymphoma, particularly in the 3^rd^ – 4^th^ year after initiating MTX therapy.

**What is known about this subject**

- Methotrexate (MTX) administration is a risk factor of malignant lymphoma for patients with rheumatoid arthritis (RA).

**What this study adds**

- We have established a clear association between the prevalence of malignant lymphoma and patients diagnosed with RA who underwent combination therapy involving MTX and biological disease-modifying antirheumatic drugs (bDMARDs). The incidence of this condition was notably higher among these patients compared to the general Japanese population.
- Our investigation revealed that combination therapy had a significantly higher risk of malignant lymphoma than MTX alone in patients with RA.
- Our findings indicate that the median time from MTX prescription to the onset of malignant lymphoma was 3.58 years (interquartile range (IQR): 2.00 – 5.34 years) for MTX-alone and 3.42 years (IQR: 1.25 – 4.92 years) for combination therapy.
- This study has provided valuable insights into the management of RA by shedding light on the incidence of malignant lymphoma in relation to different treatment approaches. Specifically, it highlights the need for increased vigilance when employing combination therapy, as it carries a higher risk of malignant lymphoma compared to MTX-alone therapy. Moreover, the study offers valuable information regarding the recommended monitoring period for detecting malignant lymphoma, thus contributing to the overall understanding and improvement of RA treatment.

## Introduction 

Methotrexate (MTX) is a key drug in the treatment of rheumatoid arthritis (RA) worldwide [1]. Biological disease-modifying antirheumatic drugs (bDMARDs) have recently been approved for RA treatment, and combination therapy with MTX and bDMARDs is recommended for patients with RA who fail to respond to MTX-alone therapy [1]. 

Malignant lymphoma associated with MTX administration is a well-known serious adverse event [2]. Additionally, inflammatory cytokines are involved in various stages of malignancy development, and bDMARDs affect the development of malignancies in patients with RA [3]. However, the incidence of malignancies with combination therapy is unknown. 

It was previously reported that combination therapy with MTX and bDMARDs for patients with RA has a higher risk of malignancies including malignant lymphoma than MTX-alone therapy, using the spontaneous adverse event reporting database [4, 5]. 

Although studies using the spontaneous adverse event reporting database can show associations between drugs and adverse events using drug safety signals, the results obtained need to be verified [6]. In addition, it is unclear how long malignancies should be monitored. Therefore, this study aimed to clarify the incidence of malignancy with combination therapy with MTX and bDMARDs in patients with RA in the working-age population and to clarify the monitoring period for malignancy using a large Japanese medical claims database. 

## Materials and methods 

### Study design 

This retrospective, observational study was conducted between April 2013 and February 2020. Patient data were obtained from the IQVIA claims database. The working-age population was defined as individuals in the age group 15 – 64 years. E360, a software-as-a-service platform (IQVIA), was used to identify patients. 

### Data collection 

The diagnosis of RA was M05 and M06 according to the International Statistical Classification of Diseases and Related Health Problems, 10^th^ edition (ICD-10). Patients diagnosed with RA were classified as those who received MTX (MTX-alone therapy) and those who received combination therapy (MTX and bDMARDs). 

Infliximab, etanercept, adalimumab, golimumab, certolizumab pegol, tocilizumab, and abatacept were classified as bDMARDs. The 12 specific target malignancy types were: stomach cancer (C16), colorectal cancer (C18 – 20), liver cancer (C22), pancreatic cancer (C25), lung cancer (C33, 34), skin cancer (C43, 44), breast cancer (C50), ovarian cancer (C56), prostate cancer (C61), kidney and urinary tract cancer (C64 – 66, C68), malignant lymphoma (C81 – 85, C96), and leukemia (C91 – 95). The observation period commenced from the first MTX prescription and ended when the patient was first diagnosed with the target malignancy, died, or could not be followed. 

### Statistical analysis 

The standardized incidence rate (SIR) was used to compare the incidence of malignancy between each group and the general Japanese population. First, the incidence of each malignancy in the target population was assumed to be the same as that in the general Japanese population. The expected number of patients who develop malignancy was calculated by summing the expected number who develop malignancy in each age group. Second, the SIR was calculated by dividing the observed number of patients who developed malignancy in the target population by the expected number who develop malignancy. The incidence rate of malignancies in the general Japanese population was targeted for 2017 [7]. 

Additionally, multivariate logistic regression analysis was performed for malignancies with a significantly high SIR. The risk of malignancy in both groups was compared. Time of onset was calculated for malignancies with significantly higher risk in either group in the multivariate analysis. Furthermore, the time of onset of malignancies in both groups was compared using the Mann-Whitney U test. Data management and analyses were performed using the Visual Analytics platform (version 1.7.0; Mathematical Systems, Inc., Tokyo, Japan) and Stata software (version 17.0; Stata Corp., College Station, TX, USA). 

## Results 

### Patient characteristics and the incidence of malignancy 

**Figure 1.**
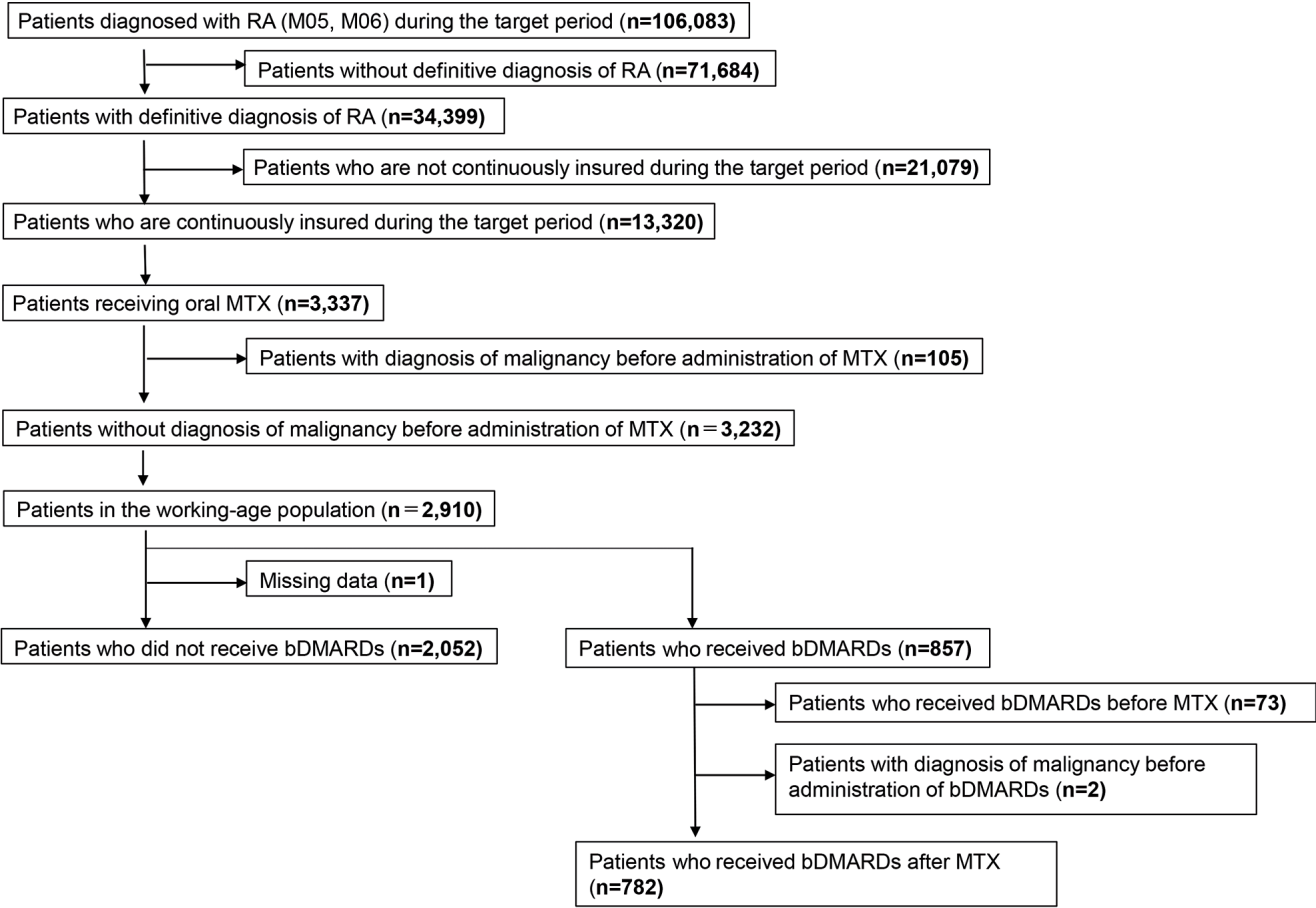
Patient inclusion criteria. RA = rheumatoid arthritis; MTX = methotrexate; bDMARDs = biological disease-modifying antirheumatic drugs.

The number of patients who met the inclusion criteria was 2,052 and 782 for MTX-alone and combination therapies, respectively (Figure 1). The detailed patient characteristics are shown in Table 1. 

In the MTX-alone therapy group, the incidence of breast cancer was remarkably higher than that in the general Japanese population (Table 2). The incidence of malignant lymphoma (SIR: 6.09, 95% confidence interval (CI): 1.58 – 10.61) was significantly higher than that in the general Japanese population. In the combination therapy group, the incidence of malignant lymphoma (SIR: 20.86, 95% CI: 8.53 – 33.19) was significantly higher than that in the general Japanese population (Table 3). 

### The risk and the time of onset of malignancy in both groups 

**Figure 2.**
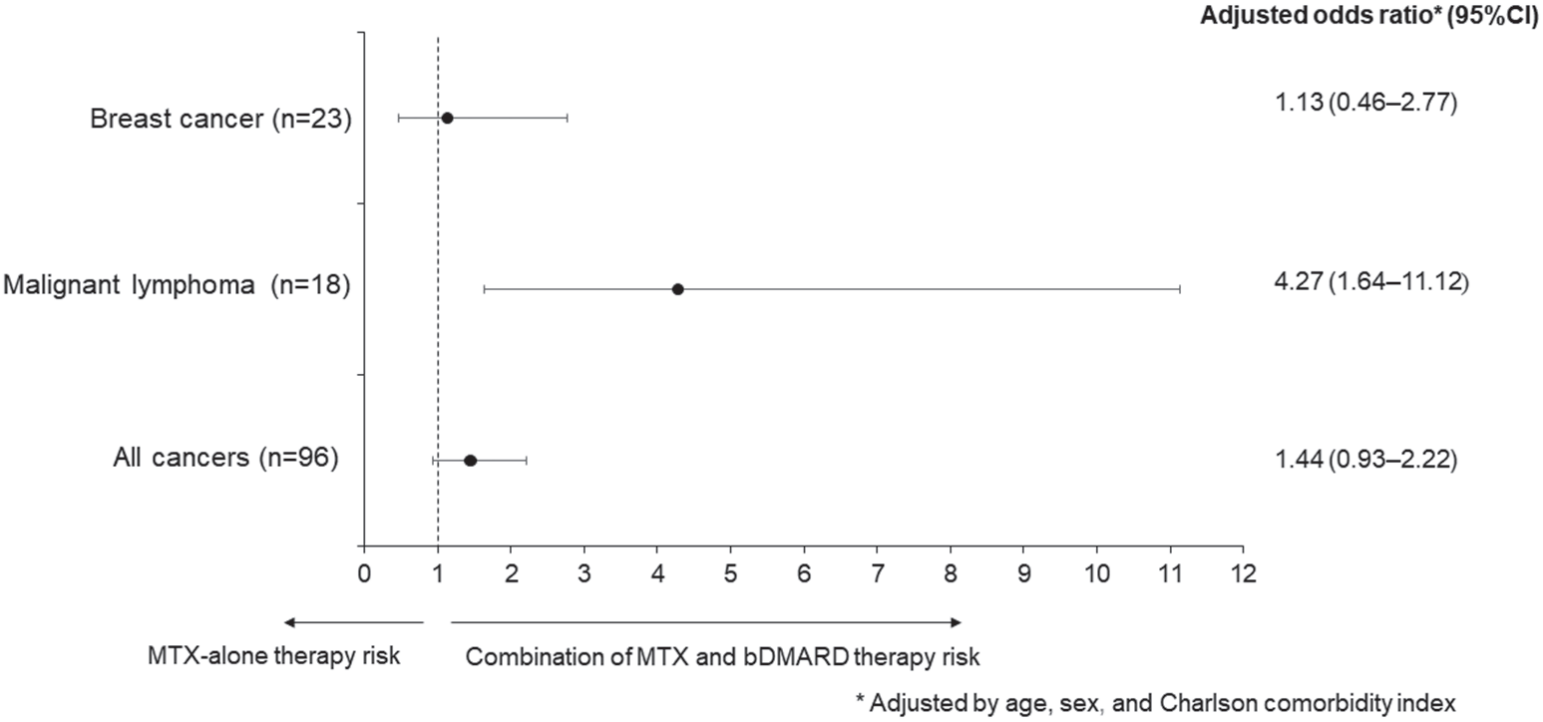
Comparison of the risk of malignancy in the MTX-alone and combination therapy groups by multivariate logistic regression analysis. MTX =methotrexate; bDMARDs = biological disease-modifying antirheumatic drugs.

**Figure 3.**
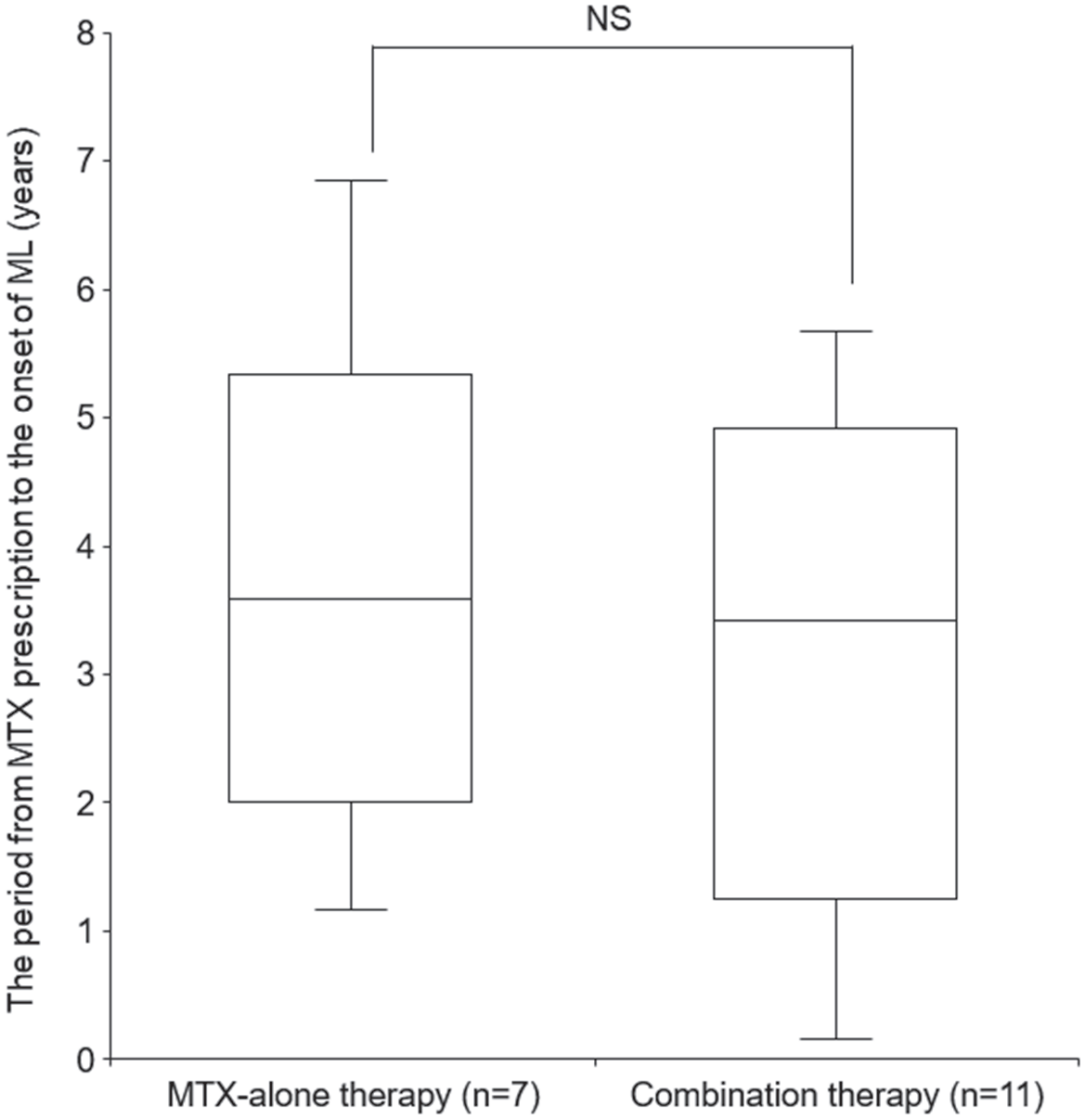
Time of onset of malignant lymphoma in the MTX-alone and combination therapy groups. “n” indicates the number of patients who developed malignant lymphoma. MTX = methotrexate; ML = malignant lymphoma.

The combination therapy group had a significantly higher risk of malignant lymphoma than the MTX-alone therapy group (adjusted odds ratio: 4.27, 95% CI: 1.64 – 11.12) (Figure 2). The median period from MTX prescription to the onset of malignant lymphoma was 3.58 years (interquartile range (IQR): 2.00 – 5.34 years) for MTX-alone therapy and 3.42 years (IQR: 1.25 – 4.92 years) for the combination therapy group, with no significant difference between both groups (p = 0.64) (Figure 3). 

## Discussion 

The incidence of malignant lymphoma was considerably higher in both groups than in the general Japanese population. Similar to previous studies [2], MTX may increase the risk of developing malignant lymphoma. In Japan, the registry of patients with RA is often used to evaluate the risk of malignancy [3]. However, many resources are required for establishing and maintaining patient registry data. In this study, we evaluated the risk of malignancy at a low cost and in a short time, using a large medical claims database. Thus, a large medical claims database could be an alternative strategy for evaluating the risk of malignancy with RA treatment. 

The combination therapy group had a markedly higher incidence of malignant lymphoma than the MTX-alone therapy group. It has been speculated that the use of bDMARDs may be related to malignancy development. Meanwhile, a high disease activity of RA is a risk factor for the development of malignant lymphoma [8]. It is possible that many patients may have a high disease activity for RA in the combination therapy group. The increased risk of malignant lymphoma with combination therapy was similar to that in the previous study using the spontaneous adverse event reporting database [4]. The results obtained using the spontaneous adverse event reporting database were validated using a Japanese medical claims database. Therefore, it is important to validate the results of one set of real-world data (RWD) using other RWDs. 

The median period from MTX prescription to the onset of malignant lymphoma was 3.58 years for MTX-alone therapy and 3.42 years for combination therapy. Therefore, it is necessary to establish a system for regularly monitoring malignant lymphoma after the initiation of MTX prescription. Particularly, strict monitoring is required in the 3^rd^ – 4^th^ year after the initiation of MTX prescription. 

This study has some limitations. First, this study was not able to consider laboratory values, disease activity of RA, and racial differences. Second, although prescription information for medications such as MTX and bDMARDs was obtained, adherence to the prescription could not be ascertained. Therefore, the possible impact of drug dosage could not be evaluated in this study. Nevertheless, the present study provided useful information to improve the prognosis of patients with RA who develop malignant lymphoma. 

## Conclusion 

In this study based on a large Japanese medical claims database, the incidence of malignant lymphoma in the combination therapy group was extensively higher than that in the general Japanese population. Special attention is therefore required for early symptoms of malignant lymphoma, particularly in the 3^rd^ – 4^th^ year after initiating MTX therapy. 

## Ethics approval 

This study was performed in accordance with the Declaration of Helsinki, and the study protocol was approved by the Ethics Committee of the Kyoto Pharmaceutical University (No. 20 – 21). As this study only used anonymized claims data, the requirement for informed consent was waived according to the requirements of the Japanese Ethical Guidelines for Medical and Health Research Involving Human Subjects. 

## Authors’ contributions 

All authors met the ICMJE authorship criteria. Ryo Inose was responsible for data analysis, study design, and preparing manuscript draft. Arisa Nakamura and Rina Omi collected data. Shujiro Takeno assisted with data interpretation and preparation of manuscript. Yuichi Muraki was responsible for study design and editing the manuscript. All authors contributed to and approved the final manuscript. 

## Funding 

This study was supported by the Japan Society for the Promotion of Science (JSPS) KAKENHI (grant numbers: 20K18865 and 21K10290). 

## Conflict of interest 

Yuichi Muraki received an honorarium from Pfizer Japan, Inc., for lecturing. The other authors declare that they have no conflict of interest. 

**Table 1.** Patient characteristics.

Characteristics	MTX-alone therapy (n = 2,052)	Combination therapy (n = 782)
Sex		
Men, n (%)	432 (21.1)	165 (21.1)
Women, n (%)	1,620 (78.9)	617 (78.9)
Age at the month of the first MTX prescription (years), n (%)		
15 – 19	14 (0.7)	9 (1.2)
20 – 24	25 (1.2)	21 (2.7)
25 – 29	40 (1.9)	27 (3.5)
30 – 34	63 (3.1)	33 (4.2)
35 – 39	139 (6.8)	63 (8.1)
40 – 44	260 (12.7)	106 (13.6)
45 – 49	334 (16.3)	136 (17.4)
50 – 54	397 (19.3)	140 (17.9)
55 – 59	441 (21.5)	148 (18.9)
60 – 64	339 (16.5)	99 (12.7)
Charlson comorbidity index		
0	55 (2.7)	21 (2.7)
1	1,351 (65.8)	491 (62.8)
≥ 2	646 (31.5)	270 (34.5)
Concomitant drugs at the month of the first MTX prescription		
Tacrolimus, n (%)	69 (3.4)	28 (3.6)
Glucocorticoid *, n (%)	650 (31.7)	289 (37.0)
Azathioprine, n (%)	8 (0.4)	0 (0)
Salazosulfapyridine, n (%)	269 (13.1)	83 (10.6)

MTX = methotrexate. *Glucocorticoids included oral prednisolone, oral betamethasone, and injectable dexamethasone sodium phosphate.

**Table 2.** Comparison of the incidence of malignancy between the MTX-alone therapy group and the general Japanese population.

	Total (n = 2,052) (6,313.2 pys)		Men (n = 432) (1,419.6 pys)		Women (n = 1,620) (4,893.7 pys)	
Type of malignancy	Case	SIR	Case	SIR	Case	SIR
Stomach	6	2.13 (0.42 to 3.83)	1	1.16 (–1.12 to 3.44)	5	3.82 (0.47 to 7.17)
Liver	1	1.22 (–1.17 to 3.61)	1	3.52 (–3.38 to 10.43)	0	N/A
Pancreas	5	5.46 (0.67 to 10.25)	1	4.11 (–3.95 to 12.17)	4	7.24 (0.14 to 14.34)
Lung	7	2.67 (0.69 to 4.64)	3	4.09 (–0.54 to 8.72)	4	2.74 (0.05 to 5.42)
Skin	2	4.01 (–1.55 to 9.56)	0	N/A	2	5.68 (-2.19 to 13.56)
Breast	16	2.63 (1.34 to 3.91)	0	N/A	16	1.68 (0.86 to 2.51)
Ovary	5	5.44 (0.67 to 10.21)	–	–	5	3.47 (0.43 to 6.52)
Prostate	2	1.41 (–0.54 to 3.35)	2	3.23 (–1.25 to 7.71)	–	–
Kidney and urinary tract	4	3.81 (0.08 to 7.54)	2	5.90 (–2.28 to 14.08)	2	4.75 (–1.83 to 11.34)
Malignant lymphoma	7	6.09 (1.58 to 10.61)	1	3.73 (–3.58 to 11.04)	6	7.23 (1.44 to 13.01)
Leukemia	4	7.84 (0.16 to 15.52)	1	7.58 (–7.28 to 22.45)	3	9.17 (–1.21 to 19.55)
Colorectal	5	1.06 (0.13 to 2.00)	1	0.81 (–0.78 to 2.39)	4	1.39 (0.03 to 2.75)
Total of the above malignancy	63	2.68 (2.02 to 3.34)	13	2.68 (1.22 to 4.14)	50	2.59 (1.87 to 3.30)

MTX = methotrexate; pys = patients-years; SIR: standardized incidence rates, N/A = not applicable. One patient was diagnosed with malignant lymphoma and colorectal cancer.

**Table 3.** Comparison of the incidence of malignancy between the combination therapy group and the general Japanese population.

	Total (n = 782) (3,273.0 pys)		Men (n = 165) (690.6 pys)		Women (n = 617) (2,582.4 pys)	
Type of malignancy	Case	SIR	Case	SIR	Case	SIR
Stomach	2	1.61 (–0.62 to 3.84)	1	2.65 (–2.55 to 7.86)	1	1.69 (–1.62 to 4.99)
Liver	1	2.78 (–2.67 to 8.23)	0	N/A	1	8.77 (–8.42 to 25.97)
Pancreas	1	2.47 (–2.37 to 7.30)	1	9.27 (–8.90 to 27.44)	0	N/A
Lung	2	1.74 (–0.67 to 4.14)	1	3.14 (–3.01 to 9.29)	1	1.54 (–1.47 to 4.54)
Skin	2	8.61 (–3.32 to 20.54)	2	36.35 (–14.03 to 86.73)	0	N/A
Breast	7	2.39 (0.62 to 4.15)	0	N/A	7	1.52 (0.39 to 2.64)
Ovary	0	N/A	–	–	0	N/A
Prostate	1	1.68 (–1.61 to 4.96)	1	3.86 (–3.70 to 11.42)	–	–
Kidney and urinary tract	1	2.09 (–2.01 to 6.19)	0	N/A	1	5.20 (–4.99 to 15.39)
Malignant lymphoma	11	20.86 (8.53 to 33.19)	6	48.58 (9.71 to 87.46)	5	13.07 (1.61 to 24.53)
Leukemia	1	4.09 (–3.93 to 12.11)	0	N/A	1	6.27 (–6.02 to 18.57)
Colorectal	4	1.89 (0.04 to 3.75)	1	1.81 (–1.74 to 5.35)	3	2.29 (–0.30 to 4.88)
Total of the above malignancy	33	3.07 (2.03 to 4.12)	13	6.08 (2.77 to 9.38)	20	2.19 (1.23 to 3.15)

pys = patients-years; SIR = standardized incidence rates; N/A = not applicable.
